# A Face-Aging Smoking Prevention/Cessation Intervention for Nursery School Students in Germany: An Appearance-Focused Interventional Study

**DOI:** 10.3390/ijerph15081656

**Published:** 2018-08-04

**Authors:** Titus J. Brinker, Jonas Alfitian, Werner Seeger, David A. Groneberg, Christof von Kalle, Alexander H. Enk, Felix J. F. Herth, Michael Kreuter, Claudia M. Bauer, Martina Gatzka, Janina L. Suhre

**Affiliations:** 1 Department of Translational Oncology, National Center for Tumor Diseases (NCT), German Cancer Research Center (DKFZ), 69120 Heidelberg, Germany; Christof.Kalle@nct-heidelberg.de; 2 Department of Dermatology, University Hospital Heidelberg, 69120 Heidelberg, Germany; Alexander.Enk@med.uni-heidelberg.de; 3 German Cancer Consortium (DKTK), University of Heidelberg, 69120 Heidelberg, Germany; 4 Department of Cardiology, University Hospital of Cologne, 50937 Cologne, Germany; jonas.alfitian@smail.uni-koeln.de; 5 Universities of Giessen and Marburg Lung Center, 35392 Gießen, Germany; Werner.Seeger@innere.med.uni-giessen.de; 6 Institute of Occupational Medicine, Social Medicine and Environmental Medicine, Goethe University, 60590 Frankfurt, Germany; arbsozmed@uni-frankfurt.de; 7 Center for Interstitial and Rare Lung Diseases, Pneumology and Respiratory Care Medicine, Thoraxklinik, University of Heidelberg, 69120 Heidelberg, Germany; Felix.Herth@med.uni-heidelberg.de (F.J.F.H.); kreuter@uni-heidelberg.de (M.K.); claudia.bauer@med.uni-heidelberg.de (C.M.B.); 8 Department of Dermatology and Allergic Diseases, Ulm University Hospital, University of Ulm, 89081 Ulm, Germany; martina.gatzka@uni-ulm.de; 9 Department of Pulmonary Medicine, University of Bonn, 53127 Bonn, Germany; janina.suhre@uni-bonn.de

**Keywords:** medical students, tobacco prevention, nurses, nursery schools, nursery students, nursery school students, smoking cessation, prevent smoking, school-based prevention

## Abstract

The Education Against Tobacco (EAT) network delivers smoking prevention advice in secondary schools, typically using the mirroring approach (i.e., a “selfie” altered with a face-aging app and shared with a class). In November 2017, however, the German assembly of EAT opted to expand its remit to include nursing students. To assess the transferability of the existing approach, we implemented it with the self-developed face-aging app “Smokerface” (=mixed − methods approach) in six nursing schools. Anonymous questionnaires were used to assess the perceptions of 197 students (age 18–40 years; 83.8% female; 26.4% smokers; 23.3% daily smokers) collecting qualitative and quantitative data for our cross-sectional study. Most students perceived the intervention to be fun (73.3%), but a minority disagreed that their own animated selfie (25.9%) or the reaction of their peers (29.5%) had motivated them to stop smoking. The impact on motivation not to smoke was considerably lower than experienced with seventh graders (63.2% vs. 42.0%; notably, more smokers also disagreed (45.1%) than agreed (23.5%) with this statement. Agreement rates on the motivation not to smoke item were higher in females than in males and in year 2–3 than in year 1 students. Potential improvements included greater focus on pathology (29%) and discussing external factors (26%). Overall, the intervention seemed to be appealing for nursing students.

## 1. Introduction

Most smokers start in their early adolescence when the risks of vascular disease, lung cancer, and chronic pulmonary disease seem too distant to be of concern [1]. When established, and after multiple failed attempts to quit, about two-thirds of smokers end up with tobacco-related diseases that physicians are unable to cure [2]. Despite the fact that the effectiveness of inpatient smoking cessation has been demonstrated in major trials, and despite being advocated in clinical guidelines [3], there remains a failure to deliver it in Germany [4,5,6,7,8,9].

Interestingly, whereas the prevalence of smoking tends to be lower among physicians than in the general population, the opposite is true for nurses [10,11]. This difference is mostly explained by social inequalities between these two groups, which strongly correlates with tobacco use [12,13,14,15,16]. Smoking among health professionals is not only a plausible barrier to delivering cessation advice but may also trigger both smoking initiation in nonsmokers and relapse in ex-smokers [10], consistent with the theory of planned behavior [17].

Education Against Tobacco (EAT) is a multinational network of medical students and physicians that uses a four-level approach to reduce tobacco-attributable disease. First, at the school level, volunteer medical students deliver interactive multimodal education in 14 countries to approximately 45,000 adolescents aged 11–16 years old per year. These address social competence and influence by making use of students’ smartphones with self-developed apps (e.g., the face-aging app “Smokerface”). The effectiveness of these interventions is assessed continuously in interventional studies and randomized trials [18,19,20,21,22,23,24,25], and currently more than 20,000 students and 400 medical students are involved in studies. Second, at the population level, the apps published by EAT (i.e., Smokerface and “Smokerstop”) are readily available, have been translated into the six most common spoken languages, and have been downloaded more than 500,000 times. Third, in medical education, EAT has prompted 13 of 28 medical schools in Germany to implement science-based modules into their curricula to educate medical students on delivering evidence-based smoking cessation counseling. Fourth, at a policy level, members of EAT organize themselves in physician associations for tobacco control efforts after graduation (e.g., *Ärzteverband Tabakprävention* in Germany).

Regarding educational efforts, it was decided at the German assembly of EAT in November 2017 to include nursing students as a target group for the face-aging interventions. It was anticipated that targeting this group, where the prevalence of smoking is high [10,26], would increase the impact of EAT interventions because nurses are an important vector for inpatient and outpatient smoking cessation interventions. Currently research also indicates that German females are more prone to appearance-based interventions than their male peers because appearance constitutes a larger part of their self-esteem [27].

### 1.1. Smoking Prevention/Cessation Interventions in German Nursing Schools

Literature on interventions to reduce smoking among nursing students in Germany is scarce, with only two tobacco prevention and cessation programs having been initiated and evaluated to date. The first was reported by Rapp et al. in 2006 and employed brief cessation counseling to help students quit smoking. The applied approach improved medical knowledge and promoted competence among the nurses when giving advice to smokers, but it had no effect on their own smoking habits [28]. The second was reported by Bühler et al. in a non-randomized, controlled feasibility study conducted in 12 German nursing schools (*n* = 397 students) from 2014 to 2015 [29]. Students in the intervention group engaged in a program that comprised an introductory session, a workshop, stress prevention lessons, an evidence-based smoking cessation intervention, and an action project. About one-third of nursing students who smoked participated in a cessation intervention. At six months follow-up, those who participated in the program seemed to do better in four outcome measures than those who received only an intervention booklet. The measures were their perceived descriptive, subjective, and injunctive norms toward smoking and nursing, as well as the perceived social support. However, yet again, there was no change in smoking behavior.

Programs designed to demonstrate face-aging and face-morphing have proven to be effective in settings where behavioral change is the goal, including for skin cancer [30,31,32], obesity [33], and tobacco use [34]. Desktop face-aging programs, in which an image is altered to predict future appearance, have been shown to be effective in convincing females aged 14–18 years to quit smoking, and even increased abstinence at 6 months among adults aged 18–30 years of both genders (abstinence levels of 27.5% in the intervention group and 6.3% in the control group) [35,36]. Based on this success, we took advantage of the high availability of smartphones and a teenager’s interest in appearance [27] to create the free 3D-photoaging smartphone app, which we named “Smokerface” [19,34], that can animate users’ selfies and react to touch. Two ongoing randomized trials are showing that this approach can increase motivation to abstain from tobacco use among seventh graders of both genders [18,25]. Moreover, a previous pilot study has shown that photoaging interventions have the potential to affect all three predictors of the theory of planned behavior [20]. According to this theory, subjective norms (e.g., “my friends think that smoking makes you unattractive”), attitudes (e.g., beliefs like “smoking leads to unattractiveness”), and perceived behavioral control (e.g., “I can refuse a cigarette”) influence both the intentions and behaviors of a person. Mirroring interventions appear to have a specific and strong influence on the subjective norm aspect of this theory [20].

### 1.2. Theoretical Considerations on Photoaging Interventions in Adolescence

The self-concept of appearance, which face-aging interventions harness, is the strongest predictor of self-esteem in adolescents of both genders. In the most recent publication by Baudson et al. involving a sample of 2950 adolescents from a broad range of secondary schools, it was noted that this is especially true for students from lower educational schools and girls. An explanation for the general effectiveness of such an intervention is given by the theory of planned behavior, according to which the subjective norm (i.e., “my friends think that smoking makes you unattractive”), the attitudes (consisting of beliefs, i.e., “smoking leads to unattractiveness”), and the perceived behavioral control (i.e., “I can resist if somebody offers me a cigarette”) influence both the behavioral intentions of a person and their behavior. Face-aging interventions may affect all three of these predictors, and the mirroring intervention specifically had a strong influence on the subjective norm in a recent pilot study.

In this study, we investigated whether the favorable results (how many students say it increases their motivation not to smoke; say their reaction of their classmates motivates them not to smoke or that the intervention is fun for them); of our face-aging intervention were reproducible in nursing students, and sought to determine what additional measures students believed would help prevent or reduce smoking in future interventions.

## 2. Materials and Methods

### 2.1. Study Design, Participants, and Setting

**Figure 1:**
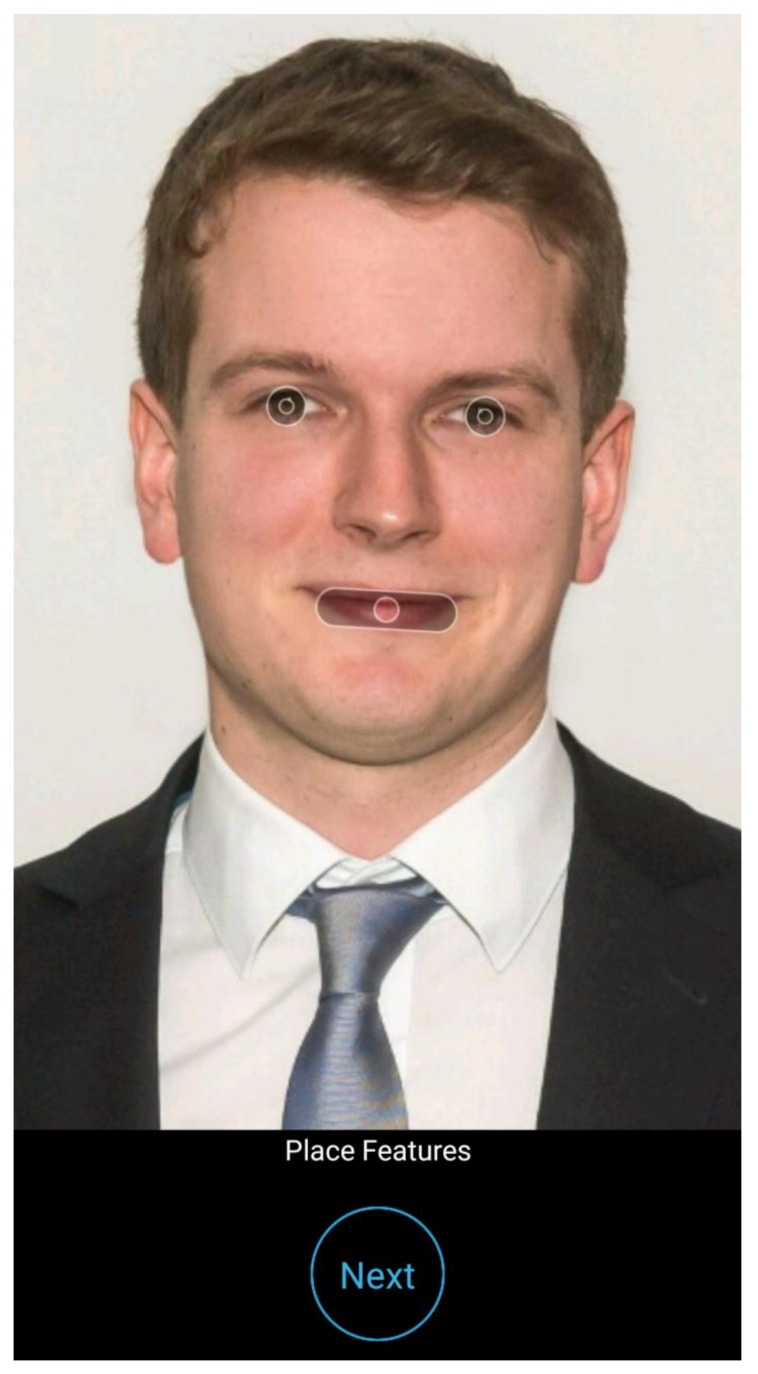
Representative initial screenshot in the Smokerface app after taking a selfie. The locations of the mouth and eyes are usually estimated correctly, but the user may adjust if needed.

**Figure 2:**
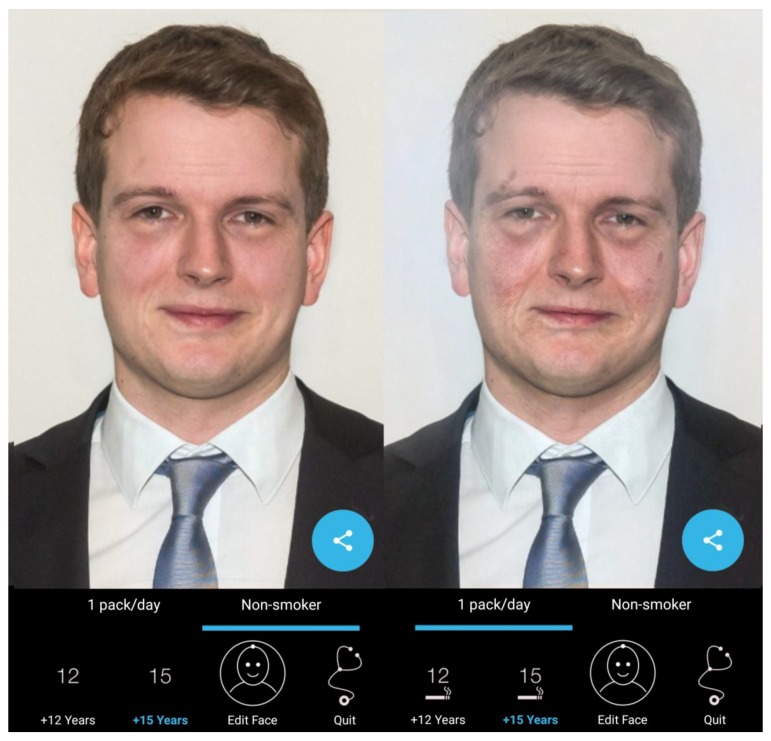
Representative animated 3D view of the effects of smoking, as displayed in the Smokerface app. Both images are shown for a Samsung Galaxy S8 device; the left image shows normal aging after 15 years and the right image shows aging with continued smoking after 15 years.

**Figure 3:**
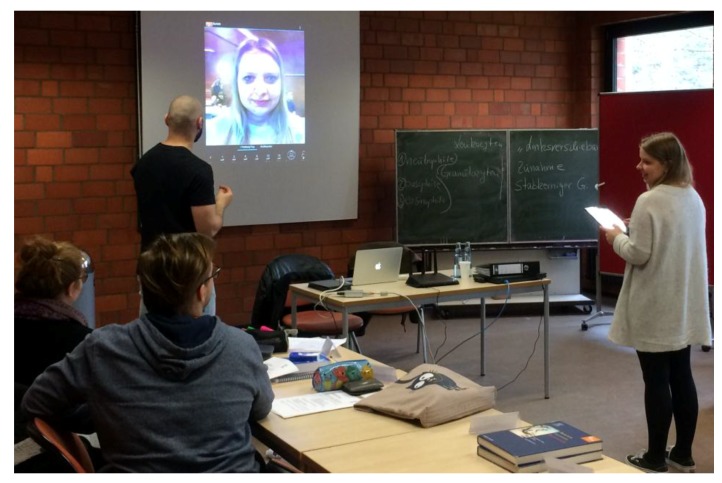
One student volunteer demonstrates the mirroring procedure in front of her class.

In this cross-sectional study, we included nursing students from six schools in Cologne and Bonn, Germany. Students were invited to participate from all three years of nurse training. Consent was assumed based on attendance and it was explained on the questionnaire that survey participation was voluntary. The ethics committee at the University of Giessen waived the necessity for an ethics approval based on the fact that the survey was anonymous, and all participants were adults. All participating nursery schools approved the procedure; the persons recognizable in Figure 1, Figure 2 and Figure 3 gave oral consent for the use of their images. The mirroring approach was used in education sessions delivered by local medical students engaged through the EAT non-profit organization. One medical student was assigned per classroom of approximately 20 students. Data were collected in March and April 2018.

### 2.2. Intervention

The mirroring intervention consists of a 30 min session using an app in the classroom, as described in our previous research [20]. The student’s altered 3D selfies on their smartphones or tablets are “mirrored” via a projector in front of their classmates and react to the user touching them (e.g., by sneezing, coughing, etc., see Video 1). The process was as follows: first, a selfie was taken and the eyes and mouth were marked by the user (Figure 1); then, in front of their peers and teachers, the students displayed their images as either a non-smoker or a smoker after intervals of 1, 3, 6, 9, 12, or 15 years (Figure 2). Multiple device displays could be projected simultaneously, a feature that was used to consolidate the altered measures with graphics (e.g., to explain how wrinkles develop). Mirroring was implemented using a 10 inch Galaxy Tab A devices (Samsung Group, Seoul, South Korea) via the AirPlay interface (Apple Inc., Cupertino, CA, USA) and the Mirroring360 app (Splashtop Inc., San José, CA, USA) for Android.

During the first 10 min, the face of one student volunteer was displayed and used to demonstrate the app’s features to the group (Figure 3). In the following 15 min, students were encouraged to try the app on their own device or on tablets that were provided for students who do not own a smartphone or did not download the app. Based on a utilization time of about 4 min per student, we anticipated needing 10 tablets to complete the mirroring intervention in a period of 25 min for a class of 40 students.

### 2.3. Post-Intervention Survey

During the last 5 min of each session, students were asked to complete an anonymous survey to assess their perceptions of the intervention. Each of the following items was measured on a 5-point Likert scale (Absolutely true, rather true, neutral, rather false, absolutely false):

(1) Change in motivation: (one item) “My 3D-selfie motivates me not to smoke”)
(2) Perceived reactions of the peer group/the subjective norm: (four items) “My classmates think I look better as a non-smoker”, “The reactions of my classmates motivate me not to smoke” and “I think I can motivate coworkers with the Smokerface App to remain abstinent/to quit”
(3) Future app use and sharing: (three items) “I plan to try this app again in the future,” “I want to install the Smokerface app on my phone,” and “I plan to show this app to other people”
(4) Global feedback: (four items) “The intervention was fun,” “I learned new benefits of non-smoking,” “The app results are realistic”; “The time of the intervention was enough for everyone to morph their face”

All of these Likert-scale-based-items were pretested and published with adolescents in the previous pilot study. Items capturing sociodemographic data as well as items relating to smoking status of different tobacco products were taken from a previous study [21,22]. We also asked students whether they believed they could use the app as a tool to motivate colleagues to quit (1) or stay abstinent (2). Lastly, we asked an open question on what the students believed would best motivate nursing students not to smoke. In our analysis, answers to this question were put in categories and the categories were ranked by frequency. All other descriptive data are reported as number/percentage (*n*/%), unless otherwise stated.

### 2.4. Data Analysis 

Descriptive analysis of data was performed with SPSS Statistics version 25 (IBM Corp., Armonk, NY, USA). No tests for significance were undertaken due to the explorative nature of the study and due to the need to take into account intracluster-correlation in a school-based study for which the sample size is insufficient.

## 3. Results

### 3.1. Participants

We included 197 students (32/16.2% male and 165/83.8% female) with a mean age of 21.88 years (median = 21 years, range = 18–40 years) from ten classes in the six participating nursing schools. Most students were in their second year of training, but the overall distribution by first, second, and third year was 19.3%, 50.2%, and 30.5%, respectively (Table 1). Passive informed consent was obtained with no drop-outs. As shown in Table 1, most smokers did so daily (23.3%), though this was higher among males than females (37.5% vs. 20.6%). The median number of cigarettes for daily smokers was 9 (range = 3.5–20), and the mean age of starting to smoke was 16.22 years (median = 16, range 11–25). Only one participant reported using an e-cigarette.

### 3.2. Perceptions of the Intervention

**Figure 4:**
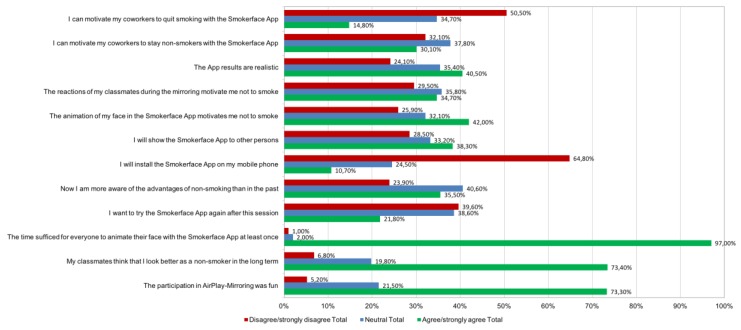
Survey results of the whole sample.

All responses from the questionnaire capturing the students’ perceptions about the intervention, are detailed in Table 2 and Figure 4. Responses were grouped so that Likert scores of 1–2 indicated “agreed” and scores of 4–5 indicated “disagreed”. In Table 2, the differences between males and females, smokers and nonsmokers, and first and second/third year students are shown; in Figure 4, an overview for the whole sample is shown. Data reported as *n*/%. Year refers to year of training. Likert scale: 1–2 = agree/strongly agree (not bold); and 4–5 = disagree/strongly disagree (bold).

#### 3.2.1. Motivation Not to Smoke

Only a minority of students disagreed or fully disagreed that their own animated selfie (50/25.9%) motivated them not to smoke (81/42.0% agreed). The results did not vary notably for males (123/92.4% agreed and 2/1.5% disagreed) and females (164/95.9% agreed and 3/1.8% disagreed; Figure 4). Generally, however, more females than males, more second/third year than first year students, and more nonsmokers than smokers agreed that the intervention had an effect (Table 2).

#### 3.2.2. Perceived Subjective Norm during the Intervention

Most students agreed that their classmates preferred their outward appearance as nonsmokers (*n* = 141/73.4%) and that their classmates’ reactions to the 3-D selfie motivated them not to smoke (*n* = 67/34.7%). Again, agreement rates were higher overall in females, more experienced students, and nonsmokers (Table 2). Only a minority of nursery students believed that the app would help coworkers either stay as nonsmokers (59/30.1%) or quit (29/14.8%). These results differed between smokers and nonsmokers. Overall, older students were more likely to perceive the tool as successful for prevention, with barely any differences between females and males.

#### 3.2.3. App Reuse and Sharing

Only a minority of students indicated that they would use the app again (43/21.8%) or would keep it on their phone (21/10.7%). However, a large proportion would show the app to other people (75/38.3%). Again, agreement rates were higher overall in females, more experienced students, and nonsmokers (Table 2).

#### 3.2.4. Global Feedback

A large proportion of participants perceived the intervention to be fun (143/73.3%; Figure 4) and learned new benefits of non-smoking (*n* = 70/35.3% agreement vs. *n* = 47/23.9% disagreement; Figure 4).

Only 79/40.5% thought the app results were realistic while almost all participants agreed that the time for everyone to take a selfie with the app was sufficient 191/97.0%. Details on subgroups may be found in Table 2.

#### 3.2.5. How Can Nursery Students Best Be Motivated Not to Smoke?

We received a total of 92 suggestions about how best to motivate nursing students not to smoke. These were grouped into five major categories and ranked by frequency. The following general results were obtained:

- the need for a stronger focus on pathologies in the prevention curriculum (*n* = 27);
- the need to change of external factors should be discussed (*n* = 24; e.g., the need for a lower nurse to patient ratio at work, stress reduction, and a replacement for smoking breaks to be used by non-smokers; interestingly, these breaks were described as a major motivation for starting to smoke at work);
- interventions should take place earlier, such as early in secondary school *n* = 8);
- prevention programs do not help and/or that it was impossible to prevent smoking in nursing students (*n* = 7);
- the advantages of quitting should be discussed and quitting advice should be offered (*n* = 6);
- nobody should interfere with personal decisions to smoke (*n* = 5); and
- finally, there were several non-categorizable statements (*n* = 15), including Smokerface, emphasizing that quitting saves money, placing a stronger focus on smokers instead of nonsmokers, giving more motivation to quit, recommending prevention programs, and some statements of “I do not know.”

## 4. Discussion

### 4.1. Principal Considerations

Mobile apps have been evaluated and optimized for use in smoking cessation [37,38,39,40,41,42,43,44,45,46,47,48,49,50,51,52,53,54,55,56,57,58,59,60], but few randomized trials have been completed. In the school setting, we identified no completed randomized trials and few app-based studies beyond our own research [34,36]. Mobile phones provide the potential to address students in an age-appropriate manner with familiar technology and activities from everyday life (i.e., taking a selfie) [61,62,63,64,65]. Another issue is that the prevalence of smoking is high among nurses [10,26]. Despite nurses being an important vector for inpatient and outpatient smoking cessation interventions, the literature on smoking prevention/cessation programs is very scarce for this target group.

### 4.2. Interpretation of Likert-Scale-Measurements from This Study

Our results indicate that face-aging interventions for tobacco prevention or cessation are less appealing in an older target group of undergraduates with a high smoking prevalence, particularly in the first year of training, when compared with seventh graders [20]. This finding is consistent with the theory of planned behavior [66]. Agreement rates were considerably lower for nursing students (81/42.0%) compared with seventh graders (79/63.2%) concerning their motivation not to smoke after seeing their own animated selfies (Table 3). More nursing students who smoked also disagreed (23/45.1%) than agreed (12/23.5%) with this item. In addition, less nurses indicated that they learned new benefits of non-smoking (agreement rates: 35.3% vs. 64.8%; Table 3). Nevertheless, the intervention was perceived as more enjoyable than in the adolescent target group (agreement rates: 73.3% vs. 61.6% in grade seven). Overall, the intervention appeared to motivate nursing students, especially female nonsmokers and those in the last years of their training.

The only caveat to this was that the sessions were viewed as considerably less convincing by nursing students when compared with seventh graders. This final point, in particular, needs to be complemented by qualitative feedback and a randomized trial.

### 4.3. Interpretation of the Qualitative Feedback

The requested focus on pathology and health aspects has been shown to be ineffective at preventing smoking uptake among secondary school students [67,68]. However, in adults, randomized trials have shown significant beneficial effects following messages that focus on health aspects. Examples of this are the effectiveness of the Centers for Disease Control and Prevention “Tips from a Former Smoker” campaign [69,70,71] and of the warning labels on cigarette packs, as implemented in Germany [72,73]. Despite this, there appears to be a paradox among nursing students who, although confronted daily by patients suffering from tobacco-attributable disease, still have a high smoking prevalence. This suggests that additional teaching on pathology would make little difference to their smoking behaviors. The second most commonly mentioned option for improving tobacco prevention was to focus on the working environment. Indeed, the fact that smokers can take more socially accepted breaks than nonsmokers was viewed as encouraging smoking behavior. A change in policies for taking breaks could be highly effective and will be addressed in a future curriculum.

Although studies have struggled to show effectiveness in a range of groups [67], it is untrue that prevention programs are ineffective or that programs targeting adolescents are most effective [28,29]. Our data, and those from previous research, indicate that targeting nursing students could be effective, with the potential for small incremental gains to major effects in the long term. Finally, the request for a stronger focus on the benefits of quitting and on how to quit should be considered reasonable given that current smokers are often aware that the habit is unhealthy but are addicted and need help with quitting. The focus on showing a problem without offering a solution is a valid criticism of the curriculum.

### 4.4. Implications for Future Research

It seems that the mirroring approach alone is important, but unlikely to be sufficient when used alone, for interventions that seeks to influence the prevalence of smoking among nursing students. Therefore, EAT plans to develop follow-up interventions to offer advice on quitting and tackling external factors (e.g., breaks for nonsmokers) through interactive exchange with nursing students.

### 4.5. Limitations

Our results stem from anonymous self-reports via questionnaires completed after the intervention. While anonymity decreases social desirability bias in self-reports, they remain less objective than externally measurable markers, particularly when recording the smoking history (e.g., cotinine saliva or carbon monoxide testing). In addition, cross-sectional data preclude any meaningful statement of effectiveness.

## 5. Conclusions

Based on these preliminary data, our app-based mirroring intervention appears to motivate nursing students, especially female nonsmokers in the final years of training, but is perceived to be considerably less convincing in this group when compared with seventh graders. Based on the qualitative feedback, EAT aims to develop a more comprehensive curriculum to be rolled out in all 80 medical schools participating in the program.

## Figures and Tables

**Table 1:** Non-smokers and smokers (including frequency) by gender and year of training.

	Do You Smoke?		How Often Do You Smoke?				Sum
	Yes	No	Daily	Not Daily, But at Least Once A Week	Less than Once A Week	Not at All	
Total	52/26.4%	145/73.6%	46/23.3%	6/3.0%	15/7.6%	130/66.0%	**197**
Male	12/37.5%	20/62.5%	12/37.5%	0/0%	2/6.3%	18/56.3%	**32**
Female	40/24.2%	125/75.8%	34/20.6%	6/3.6%	13/7.9%	112/67.9%	**165**
1st year	14/37.5%	24/62.5%	13/34.2%	2/5.3%	4/10.5%	19/50.0%	**38**
2nd year	19/19.2%	80/80.8%	18/18.1%	1/1.0%	5/5.1%	75/75.8%	**99**
3rd year	18/30.0%	42/70.0%	15/25.0%	3/5.0%	6/10.0%	36/60.0%	**60**

**Table 2:** Total results, differences by gender, smoking status and year of training.

5-Point-Likert-Scales	Total	Smokers	Nonsmokers	Year 1	Year 2–3	Male	Female
The participation on AirPlay-Mirroring was fun **^4^**: 1–2/**4–5**	143/73.3% **10/5.1%**	40/78.4% **2/3.9%**	103/71.5% **8/5.6%**	27/71.1% **1/2.6%**	116/73.9% **9/5.7%**	25/78.1% **0/0%**	118/72.4% **10/6.1%**
My classmates think that I look better as a non-smoker in the long term **^2^**: 1–2/**4–5**	141/73.4% **13/6.8%**	32/62.7% **7/13.7%**	109/77.3% **6/4.3%**	26/68.4% **5/13.2%**	115/74.7% **8/5.2%**	24/75.0% **1/3.1%**	117/73.1% **12/7.5%**
The time sufficed so that everyone could encourage their face with the Smokerface App at least once **^4^**: 1–2/**4–5**	191/97.0% **2/1.0%**	50/96.2% **1/1.9%**	141/97.2% **1/0.7%**	35/92.1% **3/7.9%**	156/98.1% **2/1.3%**	32/100% **0/0%**	159/96.4% **2/1.2%**
I want to try the Smokerface App again after this hour **^3^**: 1–2/**4–5**	43/21.8% **78/39.6%**	12/23.1% **18/34.6%**	31/21.4% **60/41.4%**	10/26.3% **14/36.8%**	33/20.8% **64/40.3%**	5/15.6% **11/34.8%**	38/23.0% **67/40.6%**
Now I am more aware of the advantages of non-smoking than in the past **^4^**: 1–2/**4–5**	70/35.5% **47/23.9%**	19/36.5% **15/28.8%**	51/35.2% **32/22.1%**	15/39.5% **12 31.6%**	55/34.6% **35/22.0%**	15/46.9% **8/25.0%**	55/33.3% **39/23.6%**
I will install the Smokerface App on my mobile phone **^3^**: 1–2/**4–5**	21/10.7% **127/64.8%**	7/13.5% **25/48.1%**	14/9.7% **102/70.8%**	3/8.1% **23/62.2%**	18/11.3% **104/65.4%**	4/12.5% **19/59.4%**	17/10.4% **108/65.9%**
I will show the Smokerface App to other persons **^3^**: 1–2/**4–5**	75/38.3% **56/28.6%**	20/38.5% **15/28.8%**	55/38.2% **41/28.5%**	13/34.2% **13/34.2%**	62/39.2% **43/27.2%**	10/31.3% **6/18.8%**	65/39.6% **50/30.5%**
The animation of my face in the Smokerface App motivates me not to smoke **^1^**: 1–2/**4–5**	81/42.0% **50/25.9%**	12/23.5% **23/45.1%**	69/48.6% **27/19.0%**	11/28.9% **12/31.6%**	70/45.2% **38/24.5%**	12/37.5% **11/34.4%**	69/42.9% **39/24.2%**
Reactions of classmates during mirroring motivates me not to smoke **^2^**: 1–2/**4–5**	67/34.7% **57/29.5%**	8/15.7% **26/51.0%**	59/41.5% **31/21.8%**	8/21.1% **15/39.5%**	59/38.1% **42/7.1%**	8/25.0% **12/37.5%**	59/36.6% **45/28%**
The app results are realistic **^4^**: 1–2/**4–5**	79/40.5% **47/24.1%**	16/31.4% **16/31.4%**	63/43.8% **31/21.5%**	12/32.4% **13/35.1%**	67/42.4% **34/21.5%**	14/43.8% **7/21.9%**	65/39.9% **40/24.5%**
With the Smokerface App I can motivate coworkers to stay nonsmokers **^2^**: 1–2/**4–5**	59/30.1% **63/32.1%**	17/33.3% **16/31.4%**	42/29% **47/32.4%**	8/21.1% **11/28.9%**	51/32.3% **52/32.9%**	11/34.4% **8/25.0%**	48/29.3% **55/33.5%**
With the Smokerface App I can motivate coworkers to top smoking **^2^**: 1–2/**4–5**	29/14.8% **99/50.5%**	4/7.8% **30/58.8%**	25/17.2% **69/47.6%**	6/15.8% **20/52.6%**	23/14.6% **79/50.0%**	4/12.5% **13/40.6%**	25/15.2% **86/52.4%**

Data reported as *n*/%. Year refers to year of training. Likert scale: 1–2 = agree/strongly agree (not bold); and 4–5 = disagree/strongly disagree (bold); ^1^ Change in. motivation; ^2^ Perceived reactions/subjective norm; ^3^ Future app-use/sharing; ^4^ Global feedback.

**Table 3:** Agreement rates among nursing students vs. secondary school students visiting grade 7.

Items	Agreement of Nursery Students	Agreement of 7th Graders
The intervention was fun	143/195, 73.3%	77/125, 61.6%
Motivated me not to smoke	81/193, 42.0%	79/125, 63.2%
Learned new benefits of non-smoking	70/198, 35.3%,	81/125, 64.8%
